# C3b/iC3b Deposition on *Streptococcus pneumoniae* Is Not Affected by HIV Infection

**DOI:** 10.1371/journal.pone.0008902

**Published:** 2010-01-26

**Authors:** Catherine Hyams, Jerry C. H. Tam, Jeremy S. Brown, Stephen B. Gordon

**Affiliations:** 1 Pulmonary Immunology, Liverpool School of Tropical Medicine, Liverpool, United Kingdom; 2 Centre for Respiratory Research, Department of Medicine, University College Medical School, Rayne Institute, London, United Kingdom; Institute of Infectious Diseases and Molecular Medicine, South Africa

## Abstract

*Streptococcus pneumoniae* is a common cause of infection in both HIV positive patients and those with complement deficiencies. We hypothesised that HIV positive individuals might exhibit reduced opsonisation of pneumococcus with complement due to reduced levels of *S. pneumoniae* specific IgG. We discovered no difference in C3 deposition on *S. pneumoniae* between HIV positive or negative individuals, and furthermore C3 deposition remained unchanged as HIV progressed towards AIDS. We found no correlation between C3 deposition on *S. pneumoniae* and CD4 cell count in HIV infected individuals. Hence we have demonstrated no failure of complement immunity in HIV positive patients.

## Introduction

*Streptococcus pneumoniae* is an important cause of morbidity and mortality in patients infected with HIV (Human Immunodeficiency Virus) and furthermore it is one of the most common bacterial pathogens to affect HIV positive adults and children [1], [2]. There are multiple reasons for increased *S. pneumoniae* linked disease in HIV positive patients, including decreased carriage, decreased CD4 (Cluster of Differentiation 4) mediated T_H_17 immunity, and altered IgG function. The polyclonal hypergammaglobulinaemia which occurs in HIV infected individuals due to non-specific activation of B lymphocytes induces a predominately IgG1 elevation [3]. However, IgG2 is often the predominant subclass of antibody produced in response to the pneumococcal polysaccharide capsule, which surrounds *S. pneumoniae* and is both the most important virulence factor for this pathogen and the target of current vaccine preparations [4], [5]. The polyclonal hypergammaglobulinaemia results in a relatively low level of capsule specific IgG in HIV infected individuals but absolute levels are not significantly different [6], [7]. IgG function has been shown to be impaired in HIV serum [8], with the mechanism for this potentially including VH3 gene repertoire deletion [9] or splenic architecture changes [10]. IgG is a potent activator of the classical complement pathway, leading to increased deposition of C3, the central complement component, on the bacterial cell surface [11]. Complement mediated immunity has been demonstrated to be important for opsonisation of the bacteria for neutrophil phagocytosis and in systemic clearance of *S. pneumoniae* infection [12]. Furthermore, individuals with chronic conditions which result in complement deficiencies, such as systemic lupus erythematosus and sickle cell anemia, also show increased incidence of invasive pneumococcal disease [13], [14]. However, individuals infected with HIV are known to have comparable levels to non-infected individuals of C3 and C4 complement components [15], [16]. It is unclear whether there is a functional deficiency in complement mediated immunity to *S. pneumoniae* in HIV but C3 deposition in HIV positive individuals could be affected by reduced complement activation due to decreased percentage of pneumococcal specific IgG or altered IgG function.

We hypothesized that the relatively high incidence of pneumococcal disease in HIV infected individuals might be due to a low level of complement deposition on the bacterial surface due to the relatively low level of specific IgG against *S. pneumoniae*. Using D39 *S. pneumoniae* as a reference strain we measured C3 deposition on the bacterial cell surface using serum samples from HIV negative and HIV positive individuals collected serially over time.

## Materials and Methods

### Samples, Patients, Ethics

Subject recruitment and sample collection Adult Malawians were recruited by advertisement and gave written informed consent to participate in a study of pulmonary immune responses to infection. This study included serum sampling and HIV testing. This study was approved by the Liverpool School of Tropical Medicine Research Ethics Committee and the College of Medicine Research Ethics Committee of the University of Malawi. Patients attended recruitment clinic when venous blood was collected and samples were transferred on ice immediately to the laboratory and centrifuged to remove the cellular pellet. Supernatant fluid and serum obtained from venous blood were stored at −80°C for future assay.

A patient cohort of 31 HIV positive and 20 HIV negative individuals was established from the Malawian cohort. The HIV infected individuals were not receiving HAART (Highly Active Antiretroviral Therapy) throughout the study, and individuals were followed over 5 years with repeat samples obtained from the same individuals as their disease progressed. These samples were collected in the period 2001–2005 before anti-retroviral therapy (ART) became available in Malawi. In 2005, all volunteers known to be HIV positive were referred to the new ART clinic together with their CD4 count and clinical files where they received expedited care. samples from HIV uninfected control subjects were selected for this study from archived samples collected during the same period (2001–2005) in the same location and by the same strategy (public advertisement) as the HIV positive samples. The cohort of subjects volunteered for wider studies of susceptibility to pneumococcal infection which included bronchoscopy and related work on blood samples.

### Bacteria

*S, pneumoniae* D39 were cultured at 37°C in 5% CO_2_ on blood agar plates or in Todd-Hewitt broth supplemented with 0.5% yeast extract to OD_580 nm_ 0.4 (approximately 10^8^ CFU/ml) and stored at –70°C in 10% glycerol as single-use aliquots.

### C3b/iC3b Binding Assay

C3b/iC3b deposition on the bacteria surface was measured using 2×10^6^ cfu of D39 *S. pneumoniae* and incubating in 10 µl 10% human serum for 20 minutes at 37°C. C3b/iC3b was measured using a previously described flow cytometry assay and fluorescein isothiocyanate (FITC) conjugated polyclonal anti-human C3 Ab (ICN) [17], [18]. Results of the complement binding assay are presented as a mean fluorescence index [FI (Fluorescent Index), proportion of positive bacteria expressed as a percentage multiplied by the geometric mean MFI] in arbitary units [12], [17], [18]. Serum samples were analysed blind to the HIV status of the individual tested.

### Statistics

Results presented as medians (IQRs) were compared using the using the Kruskal Wallis test with Dunn's multiple comparison test (multiple groups) or Mann Whitney U test (for two groups) and presented as box and whisker plots. Correlations were analysed using Pearson's Correlation Co-efficient. Data are representative of results obtained with repeated assays with four replicas per condition.

## Results

The patient cohort details are listed in Table 1.

**Table 1:** Patient cohort table.

	HIV Positive	HIV Negative	*P*-value
Number	23	15	-
Gender (M∶F)	14∶9	12∶3	0.215†
Age Mean ± SD	32.17±8.14	30.93±11.30	0.696*
CD4 Count Median (Range)	200 (0–569)	825 (456–1049)	<0.0001**
Previous Pneumococcal Disease Incidence	6	0	0.031†

*P*-values represent the results of Student t tests (*), Mann-Whitney U-Tests (**) or chi-squared tests (†).

**Figure 1:**
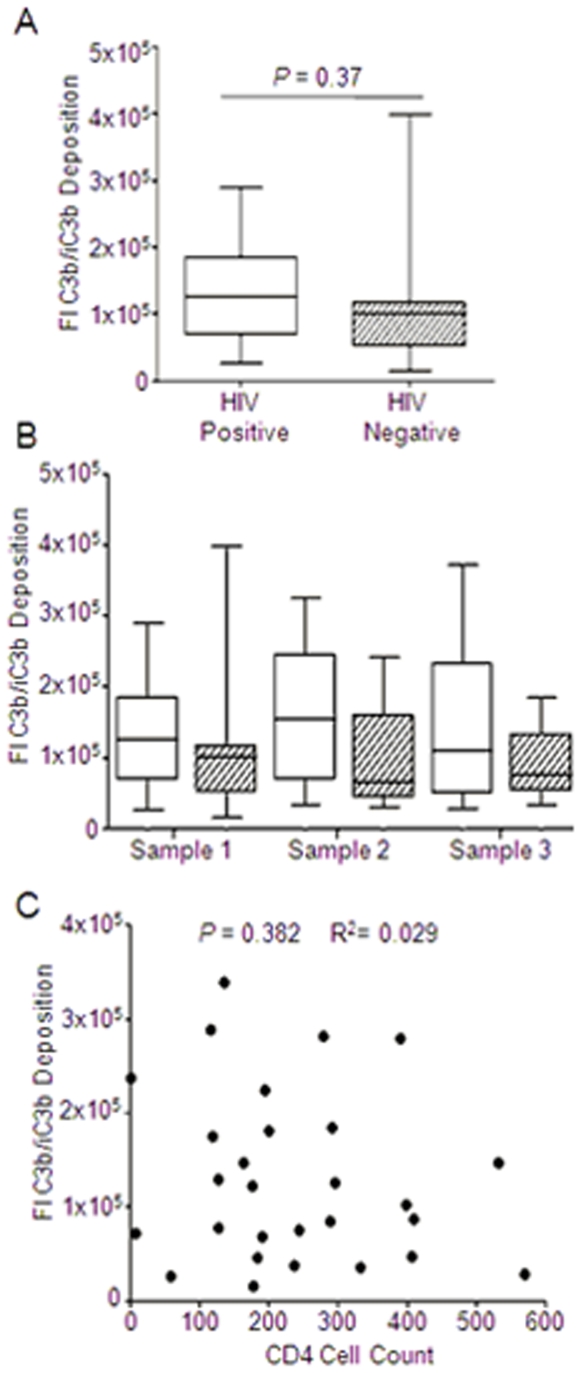
The effect of HIV infection on C3b/iC3b deposition on *Streptococcus pneumoniae*. (A) Fluorescent Index (FI) of C3b/iC3b deposition on D39 *S. pneumoniae* incubated in 20% human serum from HIV positive (open bars, n = 23) or negative individuals (slashed bars, n = 15), *P* = 0.037 Mann-Whitney U test. (B). FI of C3b/iC3b deposition on D39 incubated in 20% serum from HIV positive (open bars) or negative (slashed bars) individuals over time. Samples 1, 2 and 3 were collected sequentially from individuals. ANOVA *P* = 0.42) (C) C3b/iC3b deposition on D39 from HIV positive individuals was plotted against the CD4 cell count for that individual, Pearson's correlation co-efficient *P* = 0.382 R^2^ = 0.029).

There was no detectable difference in the ability of serum collected from HIV infected and non-infected individuals to opsonise D39 *S. pneumoniae* (Figure 1A). To investigate if the ability of serum from HIV positive patients deteriorated over time we analysed the amount of C3 deposition in serum collected from individuals with a minimum of 6 months between repeat samples as they progressed through the cohort. This was compared to serum samples collected over the same time period in the same cohort from non HIV infected patients. There was no detectable change in C3b/iC3b deposition over time in HIV negative individuals (Figure 1B). There was also no difference in C3b/iC3b deposition on *S. pneumoniae* in serum obtained from HIV positive individuals over time, suggesting no change in opsonisation as HIV progresses to AIDS.

We then examined if there was a relationship between C3b/iC3b deposition on *S. pneumoniae* and the CD4 cell count (which is a marker of HIV severity) in our HIV positive cohort (Figure 1C). We found no detectable correlation between complement deposition and CD4 count, indicating that there was no change in the ability of patients to opsonise *S. pneumoniae* in relation to the severity of HIV.

## Discussion

*Streptococcus pneumoniae* is the most common cause of bacterial pneumonia in HIV infected patients, with HIV infected children showing a disease incidence of approximately 10–100 times that of their HIV negative peers [19]. It is likely that *S. pneumoniae* disease results from an interaction between host defences and bacterial virulence factors, and a major component of host immunity to *S. pneumoniae* is the complement system [12], [18]. Complement is activated through one of three major routes, known as the classical, alternative and mannose-binding lectin pathways. Of particular importance in host immunity to *S. pneumoniae* is the classical pathway, which is activated by antibody as well other opsonins [12], [18]. Polyclonal hypergammaglobulinaemia which occurs in HIV infected individuals results in a relatively low serum capsule specific IgG but absolute antibody levels are not significantly different [6], [7]. Both the level of capsule specific IgG and the classical complement pathway (through which antibody exerts an effect on the complement system) are important to host immunity to *S. pneumoniae* [12], [20], [21]. We therefore hypothesised that a functional deficiency in the classical complement pathway due to reduced activation via IgG would lead to reduced opsonisation with C3 on the *S. pneumoniae* bacterial cell surface in HIV infected individuals compared to seronegative controls.

Our results detected no impairment in the ability of serum isolated from HIV positive individuals to opsonise a serotype 2 strain of *S. pneumoniae* compared to serum obtained from HIV negative controls. Whilst the median level of C3b/iC3b deposition for both patient populations remained approximately the same, there was increased variation in C3b/iC3b deposition on the bacterial cell surface when opsonised with serum from HIV individuals, which may perhaps be accounted for by differences in opsonising ability as HIV progresses towards AIDS (Acquired Immune Deficiency Syndrome). However there was no reduction in the relative amount of C3b/iC3b deposited on *S. pneumoniae* between serial serum samples obtained from the same individuals, indicating that there was no change in the ability of the serum to act as a opsonin as HIV disease progresses. Furthermore there was also no correlation between the CD4 count of the patient and the ability of the serum to opsonise *S. pneumoniae* with complement.

A clinically relevant depletion of C3 is seen in SLE, where in active disease without treatment, there is a depletion of 23.2% of the normal values [22]. Our study would have a 78% power to detect this. For inactive SLE, with treatment, there is a depletion to 62.8% of normal values [22], where our study would have a 27.9% power to pick up this change. In addition, there would be a hypothesised decrease in the C3 binding for HIV serum, but this was not the case, and instead there seems to be a slight increase in the binding, albeit not significant.

In conclusion, we found no detectable difference in the ability of serum obtained from HIV to opsonise *S. pneumoniae* with C3b/iC3b infected individuals compared to seronegative control individuals. We also found no reduction in deposition of C3b/iC3b on the bacterial cell surface as HIV progressed towards AIDS in our cohort. Hence, in our patient cohort there is no failure in complement deposition on pneumococcus as HIV becomes more severe and progresses to AIDS. Therefore we conclude that the polyclonal hypergammaglobulinaemia which leads to a decrease in the level of capsular specific IgG against pneumococcus does not result in a loss of function in complement deposition on *S. pneumoniae*.
